# Brain MRI findings and their association with visual impairment in young adolescents born very preterm

**DOI:** 10.1007/s00234-023-03235-5

**Published:** 2023-10-23

**Authors:** Annette Karimi, Sirkku Setänen, Eva Larsson, Gerd Holmström, Ylva Fredriksson Kaul, Olga Kochukhova, Martin Johansson, Cecilia Montgomery, Lena Hellström-Westas, Johan Wikström

**Affiliations:** 1 Department of Surgical Sciences, Neuroradiology, Uppsala University, Uppsala, Sweden; 2 Radiology Department, Uppsala University Hospital, Uppsala, Sweden; 3 Department of Pediatric Neurology, University of Turku, Turku, Finland; 4 Department of Pediatrics, Turku University Hospital, Turku, Finland; 5 Department of Surgical Sciences, Ophthalmology, Uppsala University, Uppsala, Sweden; 6 Departments of Women’s and Children’s Health, Uppsala University, Uppsala, Sweden; 7 Departments of Psychology, Uppsala University, Uppsala, Sweden

**Keywords:** Long-term follow-up, Visual impairment, White matter injury, Gestational age, Preterm birth

## Abstract

**Purpose:**

Very preterm birth increases risk for neonatal white matter injury, but there is limited data on to what extent this persists into adolescence and how this relates to ophthalmological outcomes. The aim of this study was to assess brain MRI findings in 12-year-old children born very preterm compared to controls and their association with concurrent ophthalmological outcomes.

**Methods:**

We included 47 children born very preterm and 22 full-term controls (gestational age <32 and >37 weeks, respectively). Brain MRI findings were studied in association with concurrent ophthalmological outcomes at 12-year follow-up.

**Results:**

Evans index (0.27 vs 0.25, *p*<0.001) and a proposed “posterior ventricle index” (0.47 vs 0.45, *p*=0.018) were increased in children born very preterm. Higher gestational age associated with larger corpus callosum area (β=10.7, 95%CI 0.59–20.8). Focal white matter lesions were observed in 15 (32%) of very preterm children and in 1 (5%) of full-term controls. Increased posterior ventricle index increased risk for visual acuity ≤1.0 (OR=1.07×10^11^, 95%CI=7.78–1.48×10^21^) and contrast sensitivity <0.5 (OR=2.6×10^27^, 95%CI=1.9×10^8^–3.5×10^46^). Decreased peritrigonal white matter thickness associated with impaired visual acuity (β=0.04, 95%CI 0.002–0.07).

**Conclusion:**

More white matter lesions and evidence of lower white matter volume were found in children born very preterm compared with full-term controls at 12-year follow-up. The association between larger posterior ventricle index and reduced visual acuity and contrast sensitivity suggests disturbances of the posterior visual pathway due to diffuse white matter lesions.

## Introduction

Very preterm birth increases risk for perinatal brain injury [1]. With improvements in neonatal care, severe cerebral lesions, such as intraventricular hemorrhages and cystic periventricular leukomalacia, are more seldom observed, and focus has shifted to more subtle white matter injury (WMI) [2]. White matter is particularly vulnerable to injury caused by perinatal complications such as infection, inflammation, peri- or intraventricular hemorrhage, and hypoxia–ischemia, especially in very preterm infants [3, 4]. Several studies have investigated brain magnetic resonance imaging (MRI) findings in preterm infants, revealing dilated lateral ventricles, atrophy of peritrigonal white matter, and thinning of the corpus callosum related to WMI [5–7]. Further, diffuse and extensive high signal intensities in periventricular white matter are reported secondary to WMI and preceding white matter loss [8].

Ophthalmological problems are common in children born very preterm [9, 10] and may have different underlying mechanisms. Retinopathy of prematurity (ROP) can result in various visual dysfunctions and is an important cause of childhood blindness [11]. Disturbance of the cerebral visual pathways due to WMI may cause various degrees of cerebral visual impairment in very preterm infants [9].

Structural brain MRI findings at term have been shown to predict neurodevelopmental outcomes in children born very preterm [12–14]. Perinatal diffuse WMI and long-term outcome in early adolescence have been investigated in few studies. A previous study has shown that neonatal brain MRI pathologies in infants with birth weights <2000g persist into childhood and adolescence [15]. Some other studies have reported a reduction of especially the posterior part of the corpus callosum in adolescents born preterm [16–18]. The clinical significance of long-term MRI findings, and the question if brain growth and maturation can reduce the extent of WMI merits further investigation.

The aim of the present study was to describe structural brain MRI findings in 12-year-old children born very preterm compared to full-term controls and their association with concurrent ophthalmological outcomes. The hypothesis was that children born very preterm would have more abnormal structural brain MRI findings at 12-year follow-up as compared with controls. In addition, we hypothesized that abnormal MRI findings would associate with adverse ophthalmological outcomes in children born very preterm.

## Materials and methods

### Participants

**Fig. 1:**
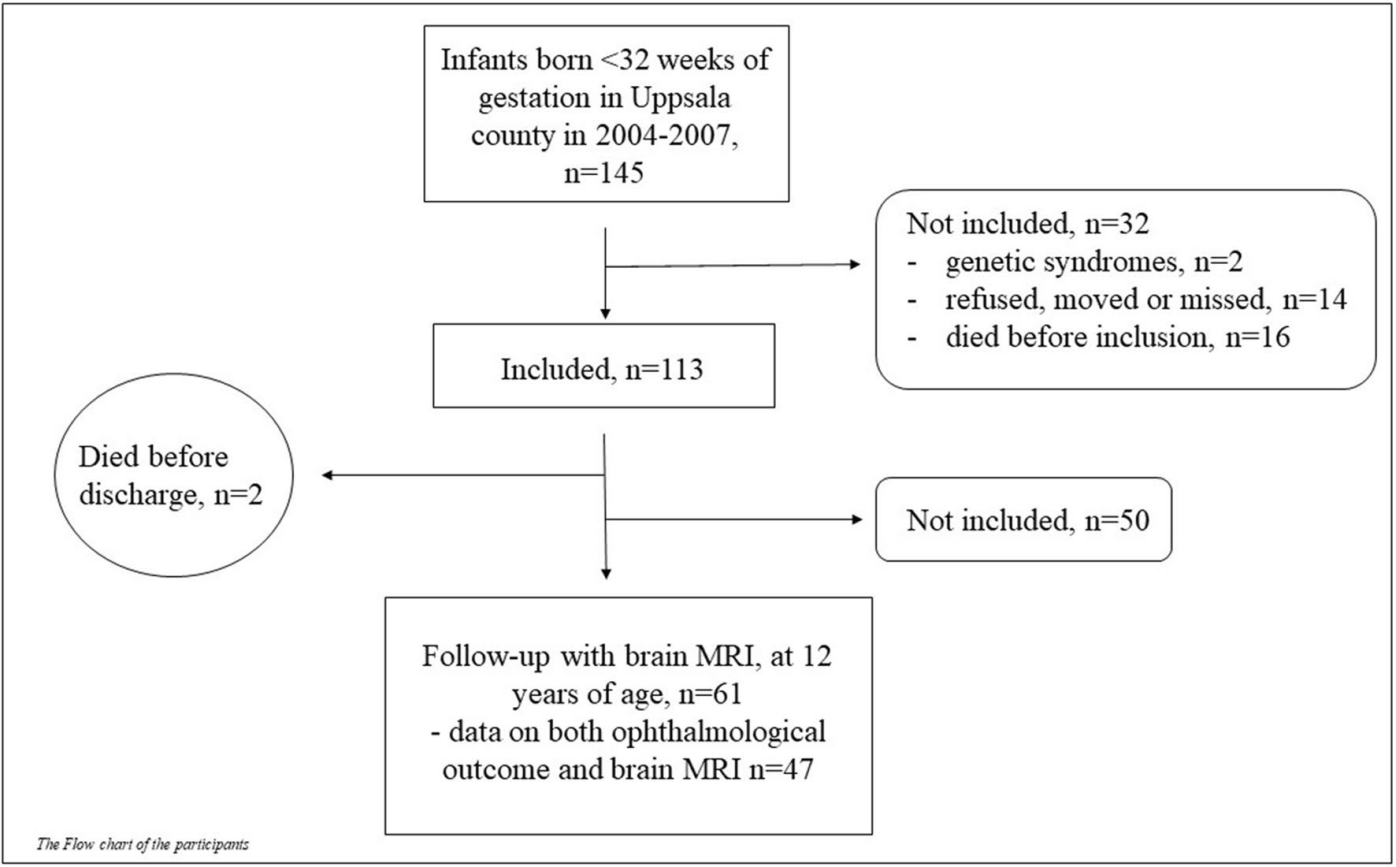
Flowchart describing the very preterm study population

This study is part of the longitudinal multidisciplinary study of visuomotor capacity in very preterm infants (LOVIS) project that is a population-based prospective follow-up study of 113 very preterm infants born <32 weeks of gestational age, in 2004–2007 in Uppsala county, Sweden [19]. Forty-seven children born very preterm and 22 full-term controls (born >37 weeks of gestational age, in 2005–2009) who underwent both brain MRI and concurrent ophthalmological assessment at 12-year follow-up were included. The mean age for MRI assessment among very preterm born children was 12.8 years, median 12.6 years, range 11.7 to 14.6 years, and among controls 13.0, 13.0, and 12.2 to 13.8, respectively. A flowchart describing the very preterm study population is shown in Figure 1. Parental written consent was obtained after written and oral information. The LOVIS study had ethical approval from the regional research ethics committee in Uppsala (Dnr 03/665 and 2016/400).

### Brain MRI

Brain MRI examinations took place between 2019 and 2022 at the Radiology Department at Uppsala University Hospital. MRI was performed without sedation using a 3 Tesla MR system (Achieva 3 T X, Philips Medical Systems, Best, Netherlands). The protocol included an axial T2-weighted sequence with TR/TE/flip angle 3381 ms/80 ms/90°, acquired resolution 0.45 × 0.45 × 4 (4 mm slice thickness), an axial 3D T1-weighted sequence with TR/TE/flip angle 8 ms/4 ms/8°, acquired resolution 1 × 1 × 1 mm (slice thickness 1 mm), and a sagittal 3D FLAIR sequence with TR/TE/TI/flip angle 4800 ms/308 ms/1650 ms/90°, acquired resolution 1.11 × 1.11 × 1.12 mm (slice thickness 1.12 mm) as well as a DWI sequence with *b* values 0 and 1000.

**Fig. 2:**
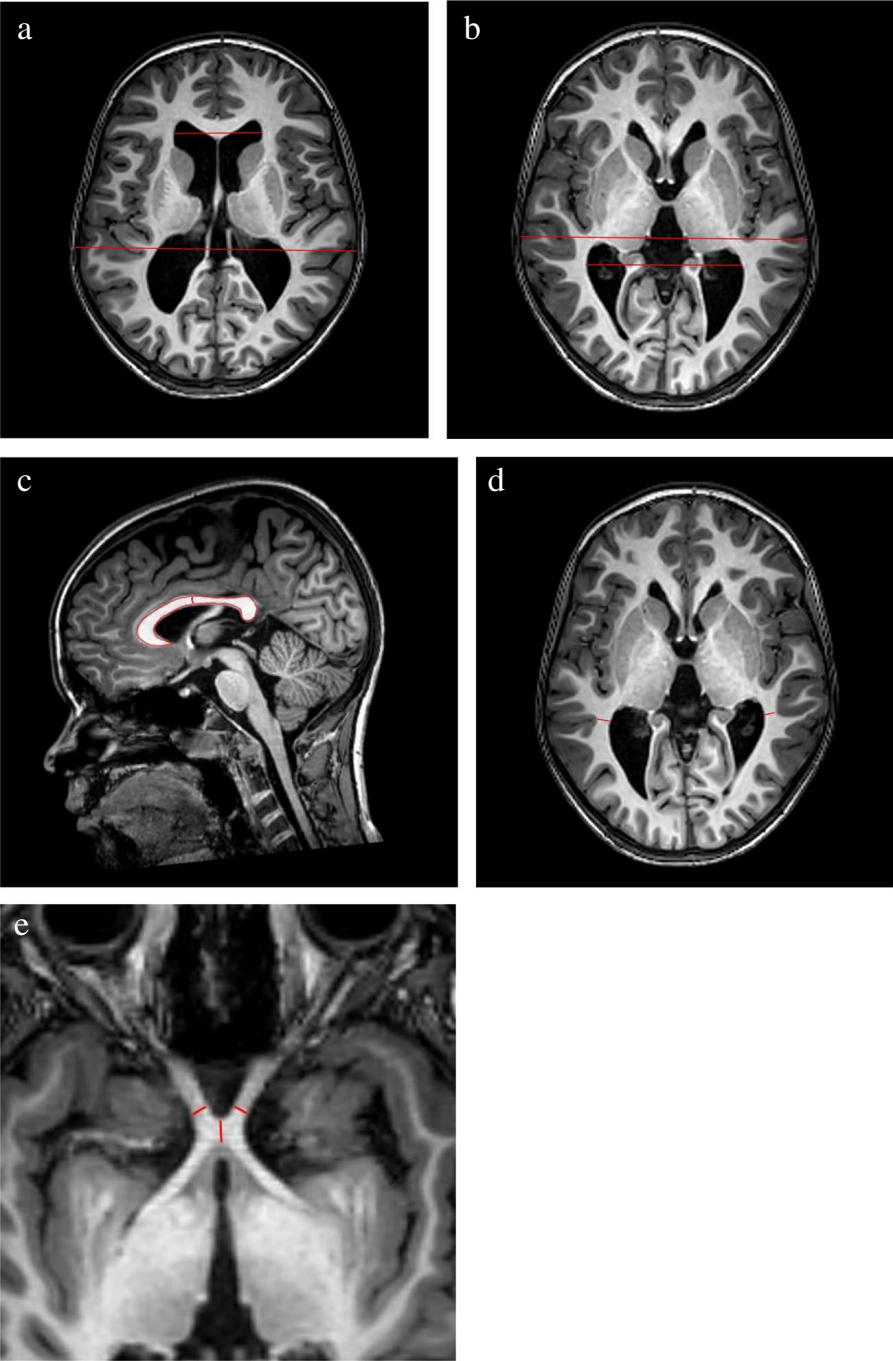
**a** The ratio of the maximum width of the frontal horns and maximal internal diameter of the skull at the same slice employed in axial MRI. **b** The maximum width of the posterior parts of the lateral ventricles (at the trigonum) were related to the maximal internal diameter of the skull. **c **The corpus callosum and the posterior part of the corpus callosum were assessed on a mid-sagittal slice. The posterior part of the corpus callosum was assessed by measuring the surface area of the posterior half. **d** The mean of the bilaterally smallest white matter part next to the trigone of the lateral ventricles was calculated in mm in order to assess the loss of white matter. **e** The thickness of the chiasma and the mean of the bilaterally optic nerve thickness was calculated in an axial T1-weighted slice

One experienced neuroradiologist (JW) and one resident (AGK) examined the scans independently of each other’s results and blinded to perinatal characteristics including gestational age. The images were assessed quantitatively and qualitatively. WMI was noted and categorized into punctate high signal lesions, confluent high signal intensities, or cystic changes. Signs of previous hemorrhage and infarcts were also assessed. In case of differing diagnosis (12 cases), the scan was re-evaluated and discussed until consensus. For quantitative measurements, the mean was computed for the two investigators results, and agreement was assessed with intraclass correlation coefficient and Bland–Altman plot. Quantitative measurements were used to assess ventricle dilatation, thinning of the corpus callosum, and white matter loss as well as the thickness of the chiasma and the optic nerves. Ventricle size was evaluated with Evans index; the ratio of maximum width of the frontal horns and maximal internal diameter of skull at the same slice employed in axial MRI (Figure 2a). To assess posterior ventricle dilatation, we introduced the “posterior ventricle index” as a new measurement for evaluation of occipital white matter reduction. Similarly to Evans index, the maximum width of the posterior parts of the lateral ventricles (at the trigonum) was related to the maximal internal diameter of skull (Figure 2b). The size of the corpus callosum was assessed by measurement of the surface area in mm^2^ on a mid-sagittal slice. The posterior part of the corpus callosum was assessed by measuring the surface area of the posterior half when divided into two parts (Figure 2c). The mean of the bilaterally smallest white matter part next to the trigone of the lateral ventricles was calculated in mm in order to assess the loss of white matter (Figure 2d). The thickness of the chiasma and the mean of the bilaterally optic nerve thickness were calculated in an axial T1-weighted slice (Figure 2e).

### Ophthalmological outcomes

All children born very preterm were screened for ROP during the neonatal period. ROP was defined according to the international classification of ROP [11], and the criteria for treatment followed the ETROP definition [20]. Mild ROP was defined as stages 1 and 2, and severe ROP was defined as stages 3–5. Ophthalmological follow-up had previously been performed at 2.5 years of corrected age [21] and at 6.5 years of chronological age [22]. In the present study, a new ophthalmological examination was performed at 12 years, all by the same orthoptist. The children used their habitual glasses at the examination. Visual acuity (Sloan letters 3 m test) was assessed, and the best-corrected binocular distance acuity (with habitual glasses) was used for calculations. Contrast sensitivity was measured binocularly at 3 m with the Lea Hyvärinen 2.5% contrast sensitivity test. The cut-offs for further analyses regarding visual acuity and contrast sensitivity and MRI data were defined as visual acuity ≤1.0 and contrast sensitivity <0.5. Manifest strabismus was assessed with the help of a cover/un-covertest at near (33 cm) and at distance (5 m). Stereo acuity was examined at near with the TNO test. A stereopsis of more than 60 s of arc was regarded as subnormal. Manifest nystagmus was recorded. Full-term controls underwent the same ophthalmological assessment.

### Statistical analysis

Differences in continuous background characteristics between study children and those who withdrew were studied using the independent samples *t*-test or Mann–Whitney *U*-test. For the categorical background characteristics, Pearson’s Chi-square test or Fisher’s exact test was used. Differences in continuous brain MRI variables between children born very preterm and full-term controls were evaluated using the independent samples *t*-test (Table 2). Differences in continuous ophthalmological variables between children born very preterm and controls were studied with Mann–Whitney *U*-test, while Pearson’s Chi-square was used for categorical variables. Associations between perinatal risk factors and brain MRI findings as well as brain MRI findings and ophthalmological outcomes were studied using generalized linear models. No child with treatment-requiring ROP was included, and therefore, no adjustment for ROP was necessary in the statistical analysis [10]. Residuals were checked to justify the analysis. Possible multicollinearity was checked; correlation coefficient ≥0.8 and/or tolerance value <0.1 and/or Phi’ and Cramers ≥0.8 was considered a sign of multicollinearity. Intraclass correlation coefficient was calculated to assess interrater agreement. The statistical analysis was performed using an SPSS version 27.0 and 28.0 (IBM SPSS Statistics, IBM Corporation, NY, USA). A two-tailed *p*-value of <0.05 was considered statistically significant.

## Results

### Perinatal characteristics

Perinatal characteristics of the included 47 children (19 girls) born very preterm are shown in Table 1. Of the participating children, none was treated for ROP, 12 had IVH grade 1, none had IVH grades 2–4, and 2 children had PVL as diagnosed on neonatal ultrasound. Apart from treated ROP, no other differences regarding perinatal characteristics between children who participated in the present study (*n*=47) and those who withdrew (*n*=66) were found (Table 1). A total of 22 full-term controls (11 girls) were included.

**Table 1:** Perinatal characteristics

Perinatal characteristics	Children who performed MRI and ophthalmological assessment (*n*=47)	Children who withdrew (*n*=66)	*p*-value
Gestational age, median (min, max), w	29.0 (24.6, 31.9)	29.4 (22.3,31.9)	0.6
Birth weight, mean (SD) g	1226.0 (333.8)	1177.8 (366.6)	0.5
Small for gestational age, *n* (%)	7 (14.9)	15 (22.7)	0.3
Female, *n* (%)	19 (40.4)	32 (48.5)	0.4
Multiple births, *n* (%)	18 (38.3)	17 (25.8)	0.2
Prenatal corticosteroids, *n* (%)	34 (72.3)	44 (66.7)	0.5
Cesarean section, *n* (%)	26 (55.3)	42 (63.6)	0.4
PVL, *n* (%)	2 (4.3)	5 (7.6)	0.7
IVH, *n* (%)	12 (25.5)	12 (18.2)	0.3
IVH grade 1, *n* (%)	12 (25.5)	7 (10.6)	
IVH grade 2, *n* (%)	0 (0)	1 (1.5)	
IVH grade 3, *n* (%)	0 (0)	3 (4.5)	
IVH grade 4, *n* (%)	0 (0)	1 (1.5)	
Severe brain injury (PVL or IVH grade 3), *n* (%)	2 (4.3)	7 (10.6 )	0.3
ROP, any, *n*(%)	9 (19.1)	23 (34.8)	0.7
ROP ≥3, *n* (%)	1 (2.1)	8 (12.1)	0.8
ROP, treated, *n* (%)	0 (0)	6 (9.1)	0.04
Sepsis, *n* (%)	11 (23.4)	11 (16.7)	0.4
Treated PDA, *n* (%)	9 (19.1)	16 (24.2)	0.5
Operated, *n* (%)	6 (12.8)	9 (13.6)	0.4
BPD, *n* (%)	10 (21.3)	15 (22.7)	0.9
NEC, *n* (%)	0 (0)	0 (0)	

### Brain MRI findings

A total of 15 (32%) children born very preterm had focal WMI: 5 (11%) had small punctate high signal lesions, and 10 (21%) had confluent high signal intensities in the white matter. No child had cystic lesions. Among full-term controls, 1 (5%) child had punctate high signal lesions. None of the focal white matter lesions were found in the optic radiation but were located in the frontal and parietal white matter regions. No control children had confluent high signal intensities or cystic lesions. There were no signs of previous hemorrhage or infarcts in either the preterm children or in full-term controls.

The mean values of Evans index, posterior ventricle index, peritrigonal white matter, corpus callosum area, and posterior corpus callosum area are presented in Table 2. Evans index (0.27 vs 0.25, *p*<0.001) and posterior ventricle index (0.47 vs 0.45, *p*=0.02) were larger in children born very preterm than in controls. We calculated lower estimates for posterior corpus callosum area in the preterm born children, but this difference did not reach statistical significance (*p*=0.08). Higher gestational age was associated with larger corpus callosum area (β=10.7, 95% CI 0.59–20.8) but not with other brain measurements in children born very preterm. The optic nerves were significantly decreased among the study children compared to the controls (4.49 vs. 4.12, *p*<0.001). No significant difference was found for the chiasma. The interrater agreement, estimated with the intraclass correlation coefficient, was 0.77, 0.54, 0.80, and 0.71 for the Evan’s index, posterior ventricle index, corpus callosum, and posterior corpus callosum, respectively. The intraclass correlation coefficient for peritrigonal white matter thickness was 0.14 and 0.20 for the right and left sides, respectively. For the chiasma, the intraclass correlation coefficient was 0.47 and for the mean of the optic nerve 0.77.

**Table 2:** Brain magnetic resonance imaging (MRI) findings in children born very preterm and full-term controls, 12-year follow-up

Brain MRI findings	Children born very preterm, *n*=47	Full-term controls, *n*=22	*p*-value
Evans index, mean (SD)	0.27 (0.02)	0.25 (0.02)	<0.001
Posterior ventricle index, mean (SD)	0.47 (0.03)	0.45 (0.02)	0.02
Peritrigonal white matter, mean (SD), mm	8.1 (1.7)	8.6 (1.5)	0.2
Corpus callosum area, mean (SD), mm^2^	563.6 (78.2)	574.3 (88.9)	0.6
Posterior corpus callosum area, mean (SD), mm^2^	266.9 (43.7)	284.5 (34.1)	0.08
Chiasma	4.59 (0.66)	4.80 (0.51)	0.08
Optic nerve	4.12 (0.40)	4.49 (0.32)	<0.001

### Ophthalmological outcome

The results of the ophthalmological examinations are presented in Table 3. There was no difference in visual acuity (*p*=0.3) and contrast sensitivity (*p*=0.3) between children born very preterm and full-term controls. Manifest strabismus was found in 6 of the preterm children but in none of the full-term controls. Further, stereo acuity was normal (≤60) in 36 (77%) and subnormal (≥120) in 11 (23.4%) children, out of which 7 (14.9%) with no stereopsis at all, while all full-term controls had normal stereo acuity (*p*<0.001). There were no children with manifest nystagmus.

**Table 3:** Ophthalmological findings in very preterm born children compared to full-term controls, 12-year follow-up

Ophthalmological findings	Children born very preterm, *n*=47	Controls, *n*=22	*p*-value
Visual acuity			
Median (min, max)	1.25 (0.8, 1.6)	1.25 (1.0, 2.0)	0.3
0.8, *n* (%)	5 (10.6)	0 (0.0)	
1.0, *n* (%)	9 (19.1)	4 (18.2)	
1.25, *n* (%)	26 (55.3)	14 (63.6)	
> 1.25, *n* (%)	7 (14.9)	4 (18.1)	
Contrast sensitivity (3 m)			
Median (min, max)	0.5 (0.25, 1.0)	0.63 (0.2, 0.8)	0.3
0.25	1 (2.1)	1 (4.5)	
0.32	3 (6.4)	0 (0.0)	
0.4	7 (14.9)	3 (13.6)	
0.5	13 (27.7)	5 (22.7)	
0.63	18 (38.3)	8 (36.4)	
0.8	3 (6.4)	5 (22.7)	
1.0	2 (4.3)	0 (0.0)	
Stereopsis			<0.001
No stereopsis *n* (%)	7 (14.9)	0 (0.0)	
240, *n* (%)	1 (2.1)	0 (0.0)	
120, *n* (%)	3 (6.4)	0 (0.0)	
60, *n* (%)	17 (36.2)	3 (13.6)	
<60, *n* (%)	19 (40.4)	19 (86.3)	
Strabismus (near and/or distance)			0.17
None, *n* (%)	41 (87.2)	22 (100.0)	
Manifest, *n* (%)	6 (12.8)	0 (0.0)	

### Associations between MRI findings and ophthalmological outcome

In children born very preterm, increased posterior ventricle index increased risk for visual acuity ≤1.0 (*p*=0.03) and contrast sensitivity <0.5 (*p*=0.005). Decreased peritrigonal white matter thickness associated with impaired visual acuity (*p*=0.04). Decreased thickness of the chiasma increased risk for subnormal stereo acuity (>60). No associations with strabismus were found. Regarding the other MRI measurements, Evans index, corpus callosum area, and posterior corpus callosum area, no significant associations with the ophthalmological outcomes were found (Table 4).

**Table 4:** Ophthalmological and MRI data in the group of 47 children born very preterm

	Visual acuity	Contrast sensitivity	Stereopsis yes/no	Stereopsis (≤ 120”)	Stereopsis (≤ 60”)	Strabismus, manifest	Strabismus, latent	Strabismus, manifest/latent	Visual acuity ≤ 0.8	Visual acuity ≤ 1.0	Contrast sensitivity < 0.5	Contrast sensitivity < 0.63
Evans index	b=0.5 (−2.0–3.0) p=0.7	*b*=0.11 (−1.7–1.9) *p*=0.9	OR=38.4 (3.6×10^−13^–4.1×10^15^) *p*=0.8	OR=71.3 (3.7×10^−12^–1.4×10^15^) *p*=0.8	OR=0.3 (3.7×10^−13^–3.1×10^11^) *p*=0.9	OR=7874 (1,4×10^−11^–4.4×10^18^) *p*=0.6	OR=3.2^−5^ (2.5×10^−16^–4.2×10^6^) *p*=0.4	OR=0 (2.3×10^−17^–6.0×10^9)^ *p*=0.6	OR=0.25 (6.4×10^−19^–1×10^15^) *p*=0.9	OR=455 (4.4×10^−9^–4.7×10^13^) *p*=0.6	OR=9.1^8^ (0–2.2×10^21^) *p*=0.2	OR=1.3 (1.1×10^−10^–1.7×10^10^) *p*=1.0
Posterior ventricle index	*b*=−1.3 (−3.2–0.6) *p*=0.18	*b*=−0.9 (−2.3–0.4) *p*=0.2	OR=33105.4 (1.7×10^−6^–6.4×10^14^) *p*=0.4	OR=618 (7.1×10^−8^–5.4×10^12^) *p*=0.6	OR=0.003 (7.4×10^−13^–1.4×10^7^) *p*=0.6	OR=4.3^6^ (6.8×10^−5^–2.7×10^17^) *p*=0.2	OR=0.007 (2.7×10^−11^–1.7×10^6^) *p*=0.6	OR= 492.1 (5.1×10^−9^–4.7×10^13^) *p*=0.6	OR=6.2×10^5^ (2×10^−6^–2×10^17^) *p*=0.3	OR=1.1×10^11^ (7.8–1.5×10^21^) *p*=0.03	OR=2.6×10^27^ (1.9×10^8^–3.5×10^46^) *p*=0.005	OR= 167024.0 (0–4.2×10^13^) *p*=0.2
White matter	*b*=0.04 (0.002–0.07) *p*=0.04	*b*=0.01 (−0.01–0.04) *p*=0.3	OR=0.7 (0.4–1.2) *p*=0.2	OR=0.7 (0.4–1.1) *p*=0.1	OR=0.9 (0.6–1.3) *p*=0.6	OR=0.8 (0.5–1.3) *p*=0.4	OR=0.9 (0.6–1.3) *p*=0.5	OR=0.7 (0.4–1.1) *p*=0.1	OR= 0.9 (0.5–1.6) *p*=0.7	OR=0.7 (0.4–1.0) *p*=0.06	OR=0.7 (0.5–1.1) *p*=0.1	OR=0.9 (0.7–1.3) *p*=0.6
Corpus callosum area	*b*=0 (0–0.001) *p*=0.3	b=0 (−7.6×10^−5^–0.001) *p*=0.09	OR=1.0 (0.99–1.0) *p*=0.5	OR=1.0 (0.99–1.0) *p*=0.4	OR=1.0 (0.996–1.0) *p*=0.3	OR=1.0 (0.993–1) *p*=0.5	OR=1.0 (0.99–1) *p*=0.7	OR=1 (0.99–1) *p*=1.0	OR=1.0 (0.99–1.0) *p*=0.8	OR=1.0 (0.99–1.0) *p*=0.4	OR=0.99 (0.98–1) *p*=0.05	OR=1.0 (0.99–1.0) *p*=0.2
Posterior corpus callosum area	*b*=0.001 (−0.001–0.002) *p*=0.3	*b*=0.001 (−2.4×10^−5^–0.002) *p*=0.06	OR=1.0 (0.99–1.0) *p*=0.3	OR=1.0 (0.99–1.0) *p*=0.2	OR=1.0 (0.994–1.0) *p*=0.2	OR=1.0 (0.992–1) *p*=0.3	OR=1 (0–98–1.1) *p*=0.7	OR=1 (0.987–1) *p*=0.6	OR=1.0 (0.98–1.0) *p*=0.8	OR=1.0 (0.99–1.0) *p*=0.5	OR=1.0 (0.97–1) *p*=0.07	OR=0.99 (0.98–1) *p*=0.2
Optic nerves	*b*=0.82 (−0.07–0.24) *p*=0.3	*b*=0.59 (−0.05–0.17) *p*=0.3	OR=3.0 (0.36–24.96) *p*=0.3	OR=1.3 (0.19–9.2) *p*=0.8	OR=0.53 (0.09–3.1) *p*=0.5	OR=4.5 (0.44–46.5) *p*=0.2	OR=0.2 (0.03–1.12) *p*=0.07	OR=0.31 (0.04–2.35) *p*=0.3	OR=14.1 (0.79–252.6) *p*=0.07	OR=4.59 (0.82–25.73) *p*=0.8	OR=2.9 (0.48–17.1) *p*=0.2	OR=1.3 (0.30–5.60) *p*=0.7
Chiasma	*b*=−0.01 (−0.1–0.08) *p*=0.8	*b*=0.009 (−0.06–0.08) *p*=0.8	OR=1.0 (0.88–1.20) *p*=0.7	OR=1.1 (0.94–1.3) *p*=0.2	OR=1.2 (1.0–1.4) *p*=0.025	OR=1.0 (0.91–1.21) *p*=0.5	OR=0.97 (0.79–1.19) *p*=0.8	OR=1.0 (0.87–1.2) *p*=0.8	OR=1.0 (0.9–1.71) *p*=0.7	OR=0.93 (0.77–1.14) *p*=0.5	OR=0.91 (0.76–1.1) *p*=0.3	OR=0.98 (0.79–1.2) *p*=0.9

## Discussion

This study evaluated structural brain MRI findings, in relation with concurrent ophthalmological findings in 12-year-old children born very preterm without severe cases of cystic white matter lesions or hemorrhagic parenchymal infarction. This included a new parameter for evaluation of posterior white matter reduction, the “posterior ventricle index.” More subtle abnormalities at brain MRI as well as ophthalmological deficiencies were found in children born very preterm compared to full-term controls, as hypothesized. Increased posterior ventricle index was found to increase risk for reduced visual acuity and contrast sensitivity and decreased peritrigonal white matter thickness associated with impaired visual acuity in children born very preterm. Decreased chiasma thickness was found to increase risk for reduced stereo acuity.

Enlargement of the lateral ventricles may represent white matter reduction secondary to injury. To focus especially on the occipital tracts including the optic radiation, we also evaluated the occipital horns with the posterior ventricle index defined as the maximum width of the occipital horns related to the maximal internal diameter of skull. In addition, we assessed peritrigonal white matter thickness. In the present study, children born preterm had an increased Evans index and posterior ventricle index compared to the children born at term, which is in line with previous studies [5, 17, 23, 24]. Skranes et al. found a dilatation of the ventricle system in 82% of adolescents born ≤1500g in 1986–1988 compared to 21% of controls at the age of 15 years. Further, ventricular enlargement was mainly seen as a focal enlargement of the occipital horns, which is consistent with our findings of an increased Posterior ventricle index [17, 24]. Griffiths et al. found ventricular dilatation in 72% among 11-year-old children born extremely preterm and in 45% among 19-year-old young adults born very preterm [5]. Aukland et al. assessed ventricle size and reported no significant group difference between 19-year-old young adults born with birth weight <2000g also in the late 1980s and controls regarding the frontal horns, while the occipital horns were wider among preterm born identifying the posterior region as especially vulnerable [23]. However, in this context, it should be mentioned that children born in the 1980s were born before the introduction of prenatal steroid treatment.

In our study, there was no significant difference in the corpus callosum area between children born very preterm and control children born at term. Posterior corpus callosum area was larger in controls than in children born very preterm, although the difference did not reach statistical significance. In children born very preterm, lower gestational age was associated with a smaller total corpus callosum area. Several previous studies have found evidence for thinning of the corpus callosum associated to preterm birth, representing a reduction in the commissural tract [16, 17, 23–25]. Especially, the posterior part of the corpus callosum seems to be affected and vulnerable for injury. Skranes et al. found thinning of the corpus callosum in 47% of 15-year-olds born ≤1500g compared to 6% of controls, reporting that mostly the posterior part was affected [17, 24]. Nosarti et al. assessed the corpus callosum size quantitatively mid-sagittal [16], similar to our measurement. They reported reduced total mid-sagittal corpus callosum area mainly due to the reduction of the posterior and mid-posterior quarters in 14–15-year-old adolescents born <33 weeks of gestation. These findings, in turn, were found to associate with adverse verbal skills in boys [16]. Aukland et al. found a reduction of the posterior third subregion of the corpus callosum adjusted with the size of the forebrain in young adults born with low birth weight [25]. The total corpus callosum area did not differ between the groups when adjusted for brain volumes. Our study strengthens previous findings regarding the occipital region being a vulnerable site with high risk for WMI in children born very preterm.

By analyzing ophthalmological outcome, we wanted to relate the structural MRI findings to the optic radiation connecting the lateral geniculate nucleus to the primary visual cortex. Visual impairment in children born preterm may be a consequence of abnormalities in the anterior visual pathway, from the eye (like ROP) to the brainstem, as well as of a disturbance of the posterior visual pathway, resulting in cerebral visual impairment [26, 27]. In the present study, children born very preterm had more often strabismus and adverse stereo acuity compared to full-term controls who all had normal stereo acuity. These findings are in line with a previous study reporting strabismus in 7% and subnormal stereo acuity in 22% of adolescents at the age of 15 years born with very low birth weight (≤1500 g) compared to 2%, respectively, 5% of the controls. Further, 9 of 17 (53%) study children with MRI abnormalities had visual dysfunction compared to 10 of 40 (25%) study children without abnormal MR findings [28]. In comparison, another study found strabismus among 16% of preterm born children at the age of 10 years [29]. Further, in a Swedish national cohort of extremely preterm infants, major eye and visual problems were found in 38% of 6.5-year-old children born extremely preterm compared with 6% of a matched control group [9]. The authors found associations between gestational age and visual problems and strabismus, respectively. Those associations disappeared when adjusted for treated ROP, indicating that treatment-requiring ROP is a strong risk factor for visual impairment. In contrast, we found associations between posterior ventricle index and visual acuity as well as with contrast sensitivity. We also found associations between white matter thickness and visual acuity, although none of the preterm born children was treated for ROP and only one child had ROP≥3.

These associations suggest that visual impairment might be a consequence of brain abnormalities in the posterior visual tract, as visualized by the posterior ventricle index and peritrigonal white matter thickness. In addition, the fact that no children treated for ROP and only one child with ROP≥3 was included in our study further supports that visual disturbances may be due to lesions of the posterior visual pathway rather than due to retinal sequelae due to ROP [27]. For a complete clinical picture of visual impairment in preterm born children, further knowledge about lesions in the posterior visual tracts is important.

A major strength of our study was its quantitative study design of the radiologic parameters, in order to improve reproducibility. Aukland et al. showed an overestimation of ventricular size and only moderate inter-observer agreement in a subjective evaluation [30]. Similarly, Kulkarni et al. reported that measurement of the ventricle size with a frontal and occipital horn ratio was superior to subjective assessment and inter-observer reliability was lower in subjective assessment [31]. In our study, a moderate to good agreement was found between the two independent observers for the quantitative evaluations of the Evan’s index, posterior ventricle index, corpus callosum, and posterior corpus callosum. On the other hand, the peritrigonal white matter thickness showed poor reproducibility. Another possible limitation of this study was its moderate follow-up rate. However, apart from treated ROP, there were no differences between participating children and those who withdrew regarding perinatal characteristics. It is unlikely that the results of the evaluated cohort are caused by selection bias, and we believe that our results are generalizable to other very preterm populations in high-income countries.

## Conclusion

Children born very preterm have more focal white matter lesions and reduced white matter compared to their full-term peers at 12 years. In addition, white matter reductions, especially in the occipital regions representing the posterior visual tract, associated with concurrent affection of visual function. To further investigate the extent of these changes, future assessments with advanced MR methods including diffusion tensor imaging and fMRI are needed.
