# Correction to: Brain MRI findings and their association with visual impairment in young adolescents born very preterm

**DOI:** 10.1007/s00234-024-03349-4

**Published:** 2024-04-10

**Authors:** Annette Karimi, Sirkku Setänen, Eva Larsson, Gerd Holmström, Ylva Fredriksson Kaul, Olga Kochukhova, Martin Johansson, Cecilia Montgomery, Lena Hellström‑Westas, Johan Wikström

**Affiliations:** 1 Department of Surgical Sciences, Neuroradiology, Uppsala University, Uppsala, Sweden; 2 Radiology Department, Uppsala University Hospital, Uppsala, Sweden; 3 Department of Pediatric Neurology, University of Turku, Turku, Finland; 4 Department of Pediatrics, Turku University Hospital, Turku, Finland; 5 Department of Surgical Sciences, Ophthalmology, Uppsala University, Uppsala, Sweden; 6 Departments of Women’s and Children’s Health, Uppsala University, Uppsala, Sweden; 7 Departments of Psychology, Uppsala University, Uppsala, Sweden

**Correction to** **:** **Neuroradiology (2024) 66:145–154**

10.1007/s00234-023-03235-5

The original article contains an error. Unfortunately, author found two minor errors in the published manuscript, which undeliberately slipped through. The corrections as follows;

- Page 8: Under the heading “Brain MRI findings”. In the sentence, “The optic nerves were significantly increased among the study children compared to the controls (4.49 vs. 4.12, *p*<0.001).” It should be corrected to decreased instead for increased.
- Page 9: Under the heading “Associations between MRI findings and ophthalmological outcome”, in the sentence “Decreased thickness of the chiasma increased risk for subnormal stereo acuity (≥120).” It should be corrected to (>60).

The original article has been corrected.

